# A Smartphone App (TRIANGLE) to Change Cardiometabolic Risk Behaviors in Women Following Gestational Diabetes Mellitus: Intervention Mapping Approach

**DOI:** 10.2196/26163

**Published:** 2021-05-11

**Authors:** Anne Lotte Potzel, Christina Gar, Jochen Seissler, Andreas Lechner

**Affiliations:** 1 Diabetes Research Group Medizinische Klinik und Poliklinik IV Klinikum der Universität München Munich Germany; 2 CCG Type 2 Diabetes Helmholtz Zentrum München Munich Germany; 3 German Center for Diabetes Research Neuherberg Germany

**Keywords:** mHealth, diabetes prevention, health behavior, cardiometabolic disease, gestational diabetes mellitus, smartphone app, intervention mapping

## Abstract

**Background:**

Gestational diabetes mellitus (GDM) is the most common complication during pregnancy and is associated with an increased risk for the development of cardiometabolic diseases. Behavioral interventions can reduce this risk, but current solutions insufficiently address the requirements for such a program. The systematic development of a scalable mobile health (mHealth) promotion program for mothers during the first years post-GDM may contribute to solving this problem.

**Objective:**

The aim of this project was to systematically plan and develop a theory- and evidence-based mHealth intervention to change cardiometabolic risk behaviors in women during the first 5 years post-GDM that meets women’s expected standards of commercial health apps.

**Methods:**

The intervention mapping steps 1 to 4 structured the systematic planning and development of the mHealth program described in this paper. Steps 1 and 2 led to a theory- and evidence-based logic model of change for cardiometabolic health. Based on this model, the prevention program was designed (step 3) and produced (step 4) in cooperation with industrial partners to ensure a high technological standard of the resulting smartphone app for the iPhone (Apple Inc). Step 4 included a user study with women during the first 5 years post-GDM once a beta version of the app (“TRIANGLE”) was available. The user study comprised 2 test rounds of 1 week (n=5) and 4 weeks (n=6), respectively. The tests included validated questionnaires on user acceptance, user logs, and think-alouds with semistructured interviews.

**Results:**

The novel TRIANGLE app is among the first self-paced smartphone apps for individual habit change in the 3 lifestyle areas of physical activity, nutrition, and psychosocial well-being. The 3 core features—a challenge system, human coaching, and a library—address 11 behavioral determinants with 39 behavior change methods to support lifestyle changes. Participants in the user study showed a high acceptance, high perceived quality, and high perceived impact of the TRIANGLE app on their health behaviors. Participants tested the app regularly, used it intuitively, and suggested improvements. We then adapted the TRIANGLE app according to the insights from the user study before the full TRIANGLE program production.

**Conclusions:**

The intervention mapping approach was feasible to plan and develop an innovative and scalable smartphone solution for women during the first 5 years post-GDM. The resulting TRIANGLE intervention has the potential to support behavior change for cardiometabolic disease prevention. However, the app needs further refinement and testing in clinical trials. Intervention mapping steps 5 (implementation plan) and 6 (evaluation plan) may support the integration of the TRIANGLE intervention into routine care.

**Trial Registration:**

German Clinical Trials Register DRKS00012736; https://www.drks.de/DRKS00012736

## Introduction

### Background

Gestational diabetes mellitus (GDM) has become a public health problem as the most common complication during pregnancy, with rising numbers globally [1]. Following GDM, women have a 10-fold higher risk for type 2 diabetes [2] and a high risk for metabolic syndrome and other cardiometabolic diseases [3,4]. Previous lifestyle interventions among women with a history of GDM who had already developed impaired glucose tolerance reduced the risk of developing type 2 diabetes by approximately 50% [5]. However, recent research highlighted the importance of starting interventions earlier, particularly in the first year postpartum [6], as the years following GDM offer a window of opportunity for preventive lifestyle interventions to prevent the progression of cardiometabolic disturbances early on [7].

### Prior Work and Proposed Solution

Qualitative research showed that lifestyle interventions within the 10 years following GDM face specific challenges such as prioritization of the family and a mother’s perceived lack of resources [8]. These challenges are reflected in the modest effects of previous behavior interventions in the first few years post-GDM [6,7]. Hence, experts call for innovative, flexible, and personalized interventions specific to the needs of women during the first few years post-GDM and the socio-ecological contexts of health behaviors [9]. These needs include a practical and accessible tool for behavior change support that is compatible with daily family life [9-11]. Mobile health (mHealth) solutions offer easy access, use in daily family life, and integrated behavior change methods [12,13]. At present, the ideal mHealth platform for women post-GDM appears to be smartphone apps since they are frequently used for health purposes by women during the first few years post-GDM [14].

### Aim of This Project

The aim of this project was to systematically plan and develop a theory- and evidence-based mHealth intervention for women during the first 5 years post-GDM that supports behavior change in several lifestyle areas. To ensure that the standards of both academia and industry were met, we decided to develop a smartphone app—the TRIANGLE app—with industrial partners using the intervention mapping protocol. Thereby, we aimed at both tackling some of the limitations from earlier prevention programs for women post-GDM [7,9] and testing the usability of the novel smartphone app with the intended users. This paper provides a detailed description of the systematic planning and development of the TRIANGLE intervention, including the user study.

## Methods

### Systematic Planning and Development of the TRIANGLE Intervention

We planned and developed the TRIANGLE intervention with the intervention mapping approach [15] as much as possible with the given resources. The process was guided by an understanding of the intervention context through the social-ecological model, systems thinking, stakeholder involvement, and ethical principles to identify and decide on the best intervention points. Further, we applied theory- and evidence-based practices using the 6 intervention mapping core processes as overarching principles. The core processes supported the use of different data sources for effective decision making by guiding us to pose a question related to the problem, brainstorm answers, review the literature, access and use theory, consider the need for new research, and define the working list of answers [15]. The 4 of the 6 intervention mapping steps that were applied in the scope of this project were a needs assessment with a logic model of the problem (step 1), a logic model of change (step 2), program design (step 3), and program production (step 4). These 4 steps provide the prerequisite for the 2 remaining steps—implementation planning (step 5) and evaluation planning (step 6)—not included in this publication.

### TRIANGLE User Study

As part of intervention mapping step 4, the TRIANGLE user study (DRKS trial registration: DRKS00012736) tested the novel TRIANGLE app in 2 test rounds with 5 or more participants of the intended target group (women in their first 5 years post-GDM) in a mixed methods design. The user study was conducted at the Medical Center of the Ludwig Maximilian University of Munich. The content of the tested TRIANGLE app was limited for the purpose of this study (Multimedia Appendix 1). Participants gave their written informed consent and signed the TRIANGLE app's privacy statement. The study was approved by the ethics committee of the Medical Faculty of the Ludwig Maximilian University of Munich.

### Study Participants

Participants were primarily recruited by phone from the patient base of the Medical Center of the Ludwig Maximilian University of Munich between mid-June and mid-July 2017. The inclusion criteria were a medical diagnosis of GDM in a recent pregnancy, 3 months to 5 years postpartum, postnatal core muscle recovery (assessed by enquiring about the completion of a 10-week course in the German health care system and a question on postnatal sporting restrictions), ownership of an iPhone 5 to 7 Plus (Apple Inc), and fluency in German. The exclusion criteria comprised age <18 years, current pregnancy, cardiopulmonary disease or restrictions in the locomotor system contraindicating a sports intervention, gastrointestinal disease contraindicating a nutrition intervention, psychiatric disease requiring therapy, other serious illness contraindicating a lifestyle intervention according to the principal investigator, and alcohol or drug abuse.

### Data Collection and Handling

Data collection comprised 2 visits (visit 1 [V1] and visit 2 [V2]) at the Medical Center of the Ludwig Maximilian University of Munich (details in Multimedia Appendix 2) with questionnaires, think-alouds with semistructured interviews (test setup in Multimedia Appendix 3), and app user logs collected during and between the 2 visits (Multimedia Appendix 4). Participants used the TRIANGLE app for a predefined period between the 2 study visits: 1 week (+ maximum 7 days) in group 1 and 4 weeks (± maximum 7 days) in group 2.

At V1, participants installed the TRIANGLE app on their iPhone and accessed their personalized account with an individual login code. They further received a Garmin vívosmart HR fitness tracker (Garmin Ltd), a step stool, and the TRIANGLE paper notepad. Participants were instructed to use the app on 5 or more days/week for the duration of the study, to conduct the fitness self-test, and to use the additional program materials. The client-server system of the TRIANGLE intervention tracked their activities on the app (Multimedia Appendix 4). The questionnaires completed during V1 included a social anamnesis, medical history, smartphone and app usage, the System Usability Scale (SUS) [16], and the user version of the Mobile Application Rating Scale (uMARS) [17]. The SUS and uMARS were also assessed during V2.

During a one-on-one think-aloud session followed by a semistructured interview, participants first received a standardized introduction video to get familiar with the test method (ie, to think aloud) before completing 30 small tasks from a standardized test protocol in the TRIANGLE app. An investigator (ALP) read aloud the tasks one by one and both observed and recorded (using audio and the iPhone screen, upon a user’s consent only) how a user installed and registered on the TRIANGLE app, attended the introduction video, and navigated through the features. Thereby, the investigator was seated outside the user’s field of vision (Multimedia Appendix 3) and noted the user’s actions in the TRIANGLE app, expressed thoughts, questions, problems, and body language in a respective checklist structured per task. If a user interrupted his or her thinking aloud, the investigator gave prompts such as, “What is on your mind?” The semistructured interviews (audio-recorded upon the user’s consent only) addressed the user’s overall impression and subjective experience with the TRIANGLE app, as well as some specifics (at V2 only) about the didactic design, content, expectations, and initial habit change. We aimed at timely insights and improvements via an explorative rapid thematic analysis before the start of the clinical pilot study. Hence, the investigator reviewed the screen and audio recordings and amended the written notes in case of a missed observation during the session instead of preparing a full transcript of each record.

Collected data were pseudonymized (4-digit code) and TRIANGLE app data were encrypted (2048-bit Secure Sockets Layer and end-to-end encryption) and stored on the server of the Medical Center of the Ludwig Maximilian University of Munich.

### Statistics

Each TRIANGLE user group contained 5 or more participants to detect approximately 80% of the issues during usability testing [18,19]. Because of the small group sizes, no statistical group comparisons were conducted. We analyzed all data in Microsoft Excel 2016 MSO (Microsoft Corporation) and Tableau Desktop 2019.3 (Tableau Software, LLC). Values are presented as counts (n) with percentages or as mean (SD). The analyses were based on all participants with the respective complete outcome measure. We excluded app activities collected during the think-aloud sessions for the app usage analyses between the 2 visits. Data from the observations during the think-alouds and the semistructured interview questions were analyzed in an explorative rapid thematic analysis with the respective numbers of participants [20,21].

## Results

### Systematic Planning and Development of the TRIANGLE Intervention

#### Step 1: Needs Assessment and Logic Model of the Problem

##### Task 1.1: Establish and Work With a Planning Group

The core team of the project (ALP and AL) is part of the Diabetes Research Group in the Medical Center of the Ludwig Maximilian University of Munich and headed the expert work groups. Expert work groups and industrial contractors covered the areas of software engineering/user interface design (n=5), sports science/personal training (n=4), nutritional science/counselling (n=3), psychological therapy/behavioral coaching (n=3), and multimedia content creation (n=2). The Diabetes Research Group is a clinical cooperation group of the Helmholtz Zentrum Muenchen and has run the 10-year Prediction, Prevention and Sub-classification of type 2 Diabetes (PPSDiab) observational study with women post-GDM since 2012 [22]. Hence, the study team gained important insights on the target group using, for example, cardiometabolic exercise testing and validated lifestyle questionnaires [23-25].

##### Task 1.2: Conduct a Needs Assessment to Create a Logic Model of the Problem (Multimedia Appendix 5)

Based on expertise and the literature [6,26,27], we defined the target group as women of reproductive age (predominantly aged 18-45 years), with ≥1 child in the household, ≥1 recent pregnancy complicated by GDM, in the extended postpartum period (maximum 5 years after delivery), and at high risk for or with cardiometabolic disturbances (as indicated by a history of GDM and the following specifications). We identified 2 main subgroups: women who were overweight with a BMI ≥23 kg/m^2^ and women with a BMI <23 kg/m^2^. We chose the threshold of BMI ≥23 kg/m^2^ previously applied to Asian populations [28] instead of the traditional threshold of BMI ≥25 kg/m^2^ [29] to define overweight since the women in the target group were younger [30] than traditional at-risk cohorts [29,31]. Therefore, weight reduction should be attempted already at a BMI ≥23 kg/m^2^. We further amended the health problem of type 2 diabetes by the following 4 main clusters of related cardiometabolic risk factors and/or disturbances to identify related risk behaviors: overweight/obesity [32,33], high blood pressure [3,34], dysglycemia [4,35], dyslipidemia [36,37], and combinations thereof [4,38]. Our related search for quality-of-life implications pointed toward little or no immediate impact of GDM on quality of life after delivery [39] (apart from some vulnerable subgroups, such as those with postpartum depression [40] or obesity [41]) versus long-term impairments in all major quality-of-life domains [42] in the presence of type 2 diabetes and/or related cardiometabolic disturbances [43,44], starting with a prediabetic state [45].

Next, we categorized the 20 main cardiometabolic risk behaviors (Textbox 1 [32,46-70]) into the lifestyle areas of physical activity, nutrition, psychosocial well-being (including sleep), and “other.“

**Cardiometabolic risk behaviors.**

**Nutrition habits**

- Excess intake of energy-dense meals or snacks [46,47]
- Excess intake of animal-derived products, especially those high in total fat/saturated fat [48]
- Excess intake of (ultra-)processed food [47]
- Excess total energy intake [32]
- Excess intake of caloric drinks such as soda [49] and alcohol [50]
- Insufficient intake of unprocessed to minimally processed food [51]
- Insufficient intake of (fresh) plant products [52]
- Insufficient intake of water, plain tea, or coffee [53,54]
- Nonadherence to recommended healthy dietary patterns and macronutrient quality [55-57]
- Insufficient control of eating behavior or emotional eating [58]

**Physical activity habits**

- Insufficient daily physical activity or sedentary behavior [59]
- Insufficient exercise or exercise intensity [60]

**Psychological and sleep habits**

- Pessimistic thinking style [61]
- Insufficient control of negative emotions [8,62]
- Poor stress management or problem solving [8,63]
- Insufficient meditation, positive emotion, optimistic thinking, or mind-body exercises [64,65]
- Insufficient enjoyable leisure time activities [66]
- Insufficient sleep-enhancing behaviors [67,68]

**Other behaviors**

- No or limited breastfeeding [69]
- Smoking [70]

The most common behavioral theories used in intervention mapping pointed to key theoretical constructs for personal determinants of behavior [15] that we used to structure the literature search for our priority population. Relevant theories included theories on automatic behavior and habits [71,72], goal setting [73], information processing/persuasive communication [74,75], process models of behavior change [76-78], and social cognitive models [79,80]. Both the behavioral theories and previous qualitative research indicated 11 main personal determinants for cardiometabolic risk behaviors in women post-GDM: habit [81], commitment [82], behavioral knowledge [83], perceived risk [84], perceived barriers to behavior [85], perceived behavioral skills [86], perceived self-efficacy [84], outcome expectations [87]/attitudes [8,81], perceived social norms [83], self-image [8], and emotional reaction to behavior [81].

##### Task 1.3: Describe the Context for the Intervention, Including the Population, Setting, and Community

The planned intervention setting was daily family life of a mother during the first 5 years post-GDM, with high demands for intervention flexibility [8] (eg, due to irregular days, financial restrictions, and/or travel during parental leave). The intervention further focused on an active relationship between health care practitioners and a woman in the first few years post-GDM to harness the momentum of the GDM diagnosis [82] and the need for personal support [88] in this locally scattered niche population. The first version of the intervention targeted behavior changes in the German cultural setting, given a high smartphone usage among mothers during the first years postpartum and numerous physical activity options.

##### Task 1.4: State Program Goals

The aim of the TRIANGLE intervention is to see a decrease in the incidence of type 2 diabetes following GDM in Germany by 30% at the 6-year follow-up after the intervention.

#### Step 2: Logic Model of Change

##### Task 2.1: State Expected Outcomes for Behavior

Similar to the Mothers After Gestational Diabetes in Australia Diabetes Prevention Program (MAGDA-DPP) trial [89], we adapted the 5 traditional behavioral end points as pursued by diabetes prevention programs (DPPs [29,31]) to women during the first 5 years post-GDM and added 2 outcomes for improved psychosocial well-being (including sleep) [58] and intervention adherence [90] (Textbox 2).

**Expected behavioral outcomes for cardiometabolic disease prevention during the first 5 years after gestational diabetes mellitus.**

- Physical activity of moderate to high intensity for ≥150 minutes/week
- Dietary fiber intake ≥15 g/1000 kcals
- Percentage of fat intake <30% of total energy intake
- Percentage of saturated fatty acid intake <10% of total energy intake
- Body weight reduction of ≥5% if BMI is ≥23 kg/m^2^; body weight maintenance if BMI is <23 kg/m^2^
- Increased psychosocial well-being and sleep, and decreased stress perception
- Intervention adherence and enhanced self-management

##### Task 2.2: Specify Performance Objectives for Behavioral Outcomes

We delineated each of the 7 expected behavioral outcomes (Textbox 2) into concrete actions that women in the first 5 years post-GDM need to perform to achieve a specific outcome. Thereby, we distinguished between preparatory one-time actions (eg, “Remove ultraprocessed food products from your home food supplies”) and habitual actions (eg, “Walk at least 10,000 steps” [daily] or “Plan your family meals for the next week” [weekly]). Our resulting list of 81 performance objectives considered the family context, flexible settings in daily life, and different entry levels with logical sequences for skill building (Textbox 3 and Multimedia Appendix 6). The high number of performance objectives allowed for tailoring of the 2 subgroups (BMI ≥23 kg/m^2^ vs BMI <23 kg/m^2^) and further individualization. Aiming primarily at long-term habit change, we excluded some of the identified cardiometabolic risk behaviors (eg, temporary behaviors, such as breastfeeding).

**Exemplary performance objectives (POs) for the first behavioral outcome: physical activity of moderate to high intensity for ≥150 minutes/week.**

- PO.1.1: Use a fitness tracker for daily step count.
- PO.1.2: Monitor the average daily step count of 1 week.
- PO.1.3: If walking less than 10,000 steps a day, decide to gradually increase daily steps to at least 10,000.
- PO.1.4: Depending on the personal starting point, walk at least 5500 steps on 3 days a week, then every day; walk at least 8000 steps on 3 days a week, then every day; and, ultimately, walk at least 10,000 steps on 3 days a week, then every day.
- PO.1.5: Disrupt longer sedentary periods every 30 minutes.
- PO.1.6: If not currently exercising, decide to initiate an exercise routine.
- PO.1.7: Conduct the fitness self-test at home.
- PO.1.8: If current exercise volume is less than 150 minutes of moderate to high intensity per week, decide to gradually increase exercise volume to reach the recommended level.
- PO.1.9: Plan own exercise regimen with health care practitioner including frequency, intensity, time, type, volume, and progression of exercise, with specific, measurable, action-oriented, realistic, timely, and self-determined goals.
- PO.1.10: Implement own exercise regimen.
- PO.1.11: Use fitness tracker for heart rate monitoring during exercise units.
- PO.1.12: Engage in active regeneration on nonexercise days.
- PO.1.13: Engage in active transportation.

##### Task 2.3: Select Determinants of Behavioral Outcomes

For optimal intervention outcomes, we formed 7 clusters of the 11 interrelated theory- and evidence-based behavioral determinants for women during the first 5 years post-GDM (Textbox 4).

**Clustered personal determinants of the performance objectives.**

- Perceived risk, behavioral knowledge, and commitment
- Perceived barriers
- Perceived skills and self-efficacy
- Outcome expectations and attitudes
- Perceived social norms
- Self-image and habits
- Emotional reaction to behavior

##### Task 2.4: Construct Matrices of Change Objectives

We crossed every performance objective with suitable determinants to uncover necessary changes for women during the first 5 years post-GDM (see example in Table 1).

**Table 1:** Change objectives (COs) for performance objective 1.12: Engage in active regeneration on nonexercise days.

Clustered personal determinants	COs
Perceived risk, behavioral knowledge, and commitment	CO.1.12.1: Get informed about the benefits of active regeneration and learn strategies for how to do it. CO.1.12.2: Acknowledge the habitual character of regeneration and the need to change environmental cues to engage in active regeneration. CO.1.12.3: Decide to engage in active regeneration on nonexercise days.
Perceived barriers	CO.1.12.4: Get informed about possible perceived barriers to engaging in active regeneration and identify personal barriers. CO.1.12.5: Get informed about possible solutions to overcome perceived barriers to engaging in active regeneration and implement the most suitable solutions. CO.1.12.6: Expect and resist hindering social pressure by family members or the wider social network when engaging in active regeneration.
Perceived skills and self-efficacy	CO.1.12.7: Express confidence in ability to engage in active regeneration or to learn how to do so. CO.1.12.8: Feel capable of noticing automaticity on regeneration days and altering cues that trigger engaging in active regeneration.
Outcome expectations and attitudes	CO.1.12.9: Expect that engaging in active regeneration leads to less aching muscles and cardiometabolic health benefits. CO.1.12.10: Feel positive about engaging in active regeneration.
Perceived social norms	CO.1.12.11: Notice that most physically fit individuals consistently engage in active regeneration and find role models in their own social network. CO.1.12.12: Notice that engaging in active regeneration does not need approval by others.
Self-image and habits	CO.1.12.13: Consistently maintain an active regeneration routine until habitual. CO.1.12.14: Identify as a healthy homemaker and role model who guides own children and partner to enjoy being active together.
Emotional reaction to behavior	CO.1.12.15: Expect initial discomfort when not used to engaging in active regeneration. CO.1.12.16: Notice that engaging in active regeneration is fun and does not translate to constraints. CO.1.12.17: Feel great about having engaged in active regeneration.

##### Task 2.5: Create a Logic Model of Change (Multimedia Appendix 7)

The logic model of the problem (intervention mapping step 1; Multimedia Appendix 5) and the matrices of change objectives (Task 2.4; Table 1) informed our logic model of behavior change for cardiometabolic health during the first 5 years post-GDM.

#### Step 3: Program Design

##### Task 3.1: Generate Program Themes, Components, Scope, and Sequence

We collected program requirements for women during the first few years post-GDM as assessed in previous qualitative studies via interviews, observations, open-ended surveys, and focus groups [8]. We then translated the requirements into the following 4 clusters of mHealth specifications: (1) 3 lifestyle areas under the umbrella of behavioral psychology, (2) family context, (3) individual content and participation, and (4) other specific needs of women during the first years post-GDM. We stressed the intertwined nature of the 3 main lifestyle areas that form the intervention modules (physical activity, nutrition, and psychosocial well-being) by using a symbolic health triangle as the logo and naming the smartphone app TRIANGLE. The branding of the TRIANGLE intervention with a challenge system reflects the challenging nature of multiple habit changes and the overarching module of behavior change. We planned to deliver all modules digitally (Multimedia Appendix 8) with a flexible dosage and sequence to account for individual differences in habit formation. Essential program components include multimedia knowledge transfer, individual behavior change support, and skill training. Additional program materials comprise a fitness wrist band, a step stool, and the TRIANGLE paper notepad.

##### Tasks 3.2 and 3.3: Choose and Translate Theory- and Evidence-Based Change Methods Into Practical Applications

In line with the intervention mapping–related taxonomy of behavior change methods [91], we selected 39 behavior change methods (Table 2) to address all specified change objectives and translated them into app features. Thereby, we focused on habit formation as shared characteristics of the 3 lifestyle areas. This resulted in the following 3 core features of the app: (1) an interactive challenge system, (2) individual human coaching, and (3) a library. An associated coaching platform mirrored these features for content management and individualization. All interactions were designed to be short in duration; for example, a participant may accept a challenge, the personal coach then motivates the participant to commit to this challenge despite the delayed health benefits, and the library provides a short article on the benefits of early commitment.

**Table 2:** Behavior change methods to address clustered personal determinants.

Clustered personal determinants	Behavior change methods
Perceived risk, behavioral knowledge, and commitment	Tailoring Chunking Advance organizers Using imagery Discussion Framing Environmental reevaluation Credible information Individualization Participation Technical assistance Persuasive communication Consciousness raising
Perceived barriers	Participatory problem solving Planning coping responses Resistance to social pressure
Perceived skills and self-efficacy	Guided practice Enactive mastery experiences Verbal persuasion Improving emotional states Self-monitoring Technical assistance Goal setting Setting graded tasks Cue altering
Outcome expectations and attitudes	Arguments Direct experience Belief selection Providing contingent rewards Elaboration
Perceived social norms	Information about others’ approval Resistance to social pressure Mobilizing social support
Self-image and habits	Counterconditioning Implementation intentions Stimulus control Early commitment Public commitment Technical assistance Reinforcement Self-reevaluation Environmental reevaluation
Emotional reaction to behavior	Self-reevaluation Improving physical and emotional states Direct experience Feedback

#### Step 4: Program Production

##### Task 4.1: Refine Program Structure and Organization

Our market analysis showed a gap between the identified requirements and current health promotion programs for women during the first 5 years post-GDM. Therefore, we decided to produce new program materials to address all identified requirements. Iterative processes helped us define the intervention modules (Multimedia Appendix 8) and the software requirements for the subfeatures of the app and the coaching platform. We prioritized the production of high-quality multimedia content as follows: 136 challenges and 133 library articles with an introduction and a medical background video, 54 videos related to physical activity (27 guided practices, 5 fitness self-tests, and 22 short tutorials), 8 psychological audio exercises, and 241 images. The challenges were mainly derived from the list of performance objectives (step 2) and contained options for tailoring and individualization. An initial paper-and-pencil questionnaire and in-app questionnaires using the Apple ResearchKit informed the individualization process (eg, according to personal preferences and goals).

##### Task 4.2: Prepare Plans for Program Materials

**Figure 1:**
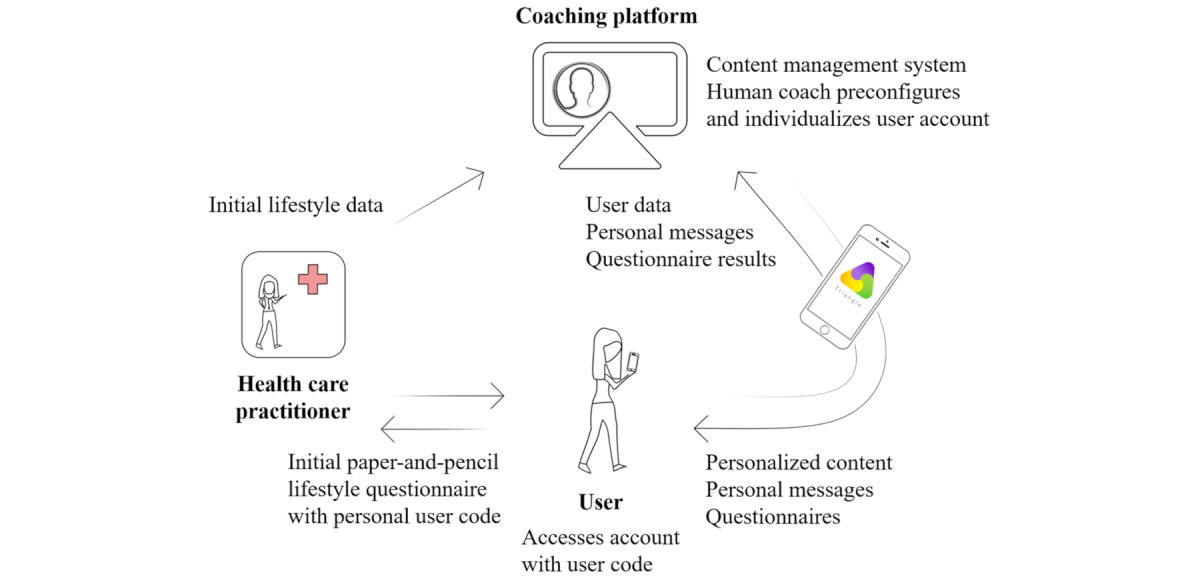
TRIANGLE app onboarding and workflow.

The content of the TRIANGLE intervention needed to match the features of the planned app. Therefore, we developed design documents and style templates for the planned content (questionnaires, challenges, and library articles) and a scheme of the work and data flow between health care practitioners, the coaching platform, and app users/the app (Figure 1). Thereby, we considered smartphone-specific issues such as users' short attention span, screen limitations, and data protection. We secured a sociocultural fit of the intervention specific to Central European women during the first few years post-GDM in the multimedia content by involving female experts of childbearing age in all work groups. Further, we briefed all lifestyle work groups on the style templates to guarantee coherence.

##### Task 4.3: Draft Messages, Materials, and Protocols

**Figure 2:**
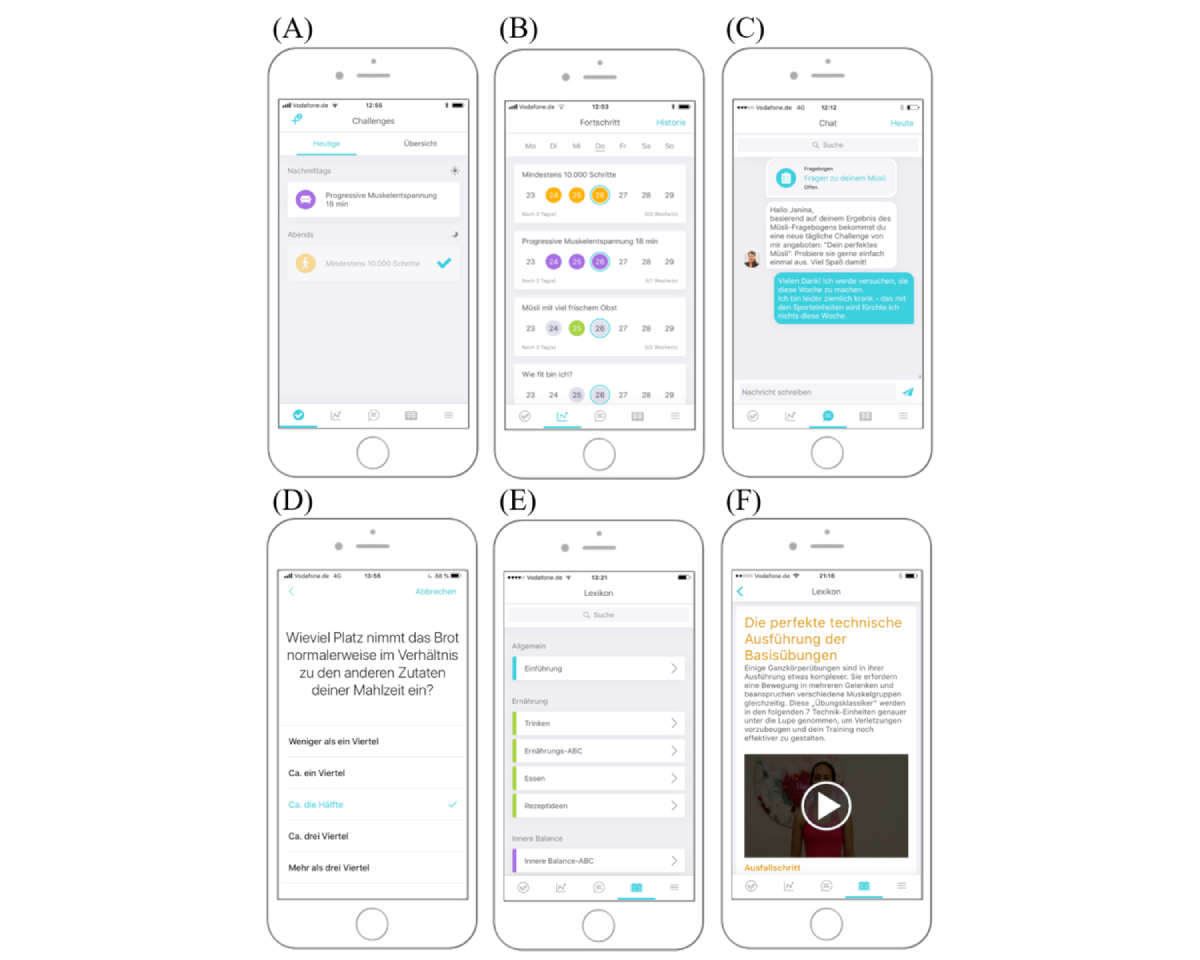
Screenshots of the TRIANGLE app's core features. Challenge system: (A) activity screen based on active challenges and (B) weekly progress visualization per challenge. Coaching: (C) chat with personal coach and (D) in-app questionnaires. Library: (E) categories per theme and (F) exemplary article.

The software programming started with mock-ups in parallel with the content development by the lifestyle work groups. Given time and budget limitations, we decided to program the first version of the app for the iOS system for iPhones only, in line with the iOS developer human interface guidelines. The TRIANGLE app's core features were realized as follows (Figure 2): the interactive and individualized challenge system contains an activity screen, self-monitoring with progress visualization, and notifications/reminders. The individual human coaching is based on positive psychology, personal text messaging with a health care practitioner, in-app questionnaires, and personalized content/feedback. The library includes different modules with multimedia content and a keyword search.

We tested the app's features after each programming cycle, with thorough alpha testing of the app before the start of the user study. We specified which type of user data would be collected to allow for tailoring and individualization while complying with the General Data Protection Regulation. Further, we implemented encryption and authentication measures to protect against data misuse by third parties. A step-by-step guide supported the registration process of a participant. Our chosen lifestyle color scheme is yellow for physical activity, green for nutrition, and purple for psychosocial well-being, while the overarching color scheme is light blue and light grey, as often used in health care [58,92,93].

##### Task 4.4: Pretest, Refine, and Produce Materials

The pretest was conducted in the scope of the TRIANGLE user study.

### TRIANGLE User Study

The demographic characteristics of the user sample are shown in Table 3.

**Table 3:** Demographic characteristics of participants in the TRIANGLE user study.

Characteristics		Total (n=11)	Group 1 (n=5)	Group 2 (n=6)
Age (years), mean (SD)		36.6 (2.2)	35.4 (1.5)	37.7 (2.3)
Partner status: with partner, n (%)		10 (90.9)	5 (100)	5 (83.3)
**Number of children, n (%)**				
	1	5 (45.5)	3 (60.0)	2 (33.3)
	2	6 (54.5)	2 (40.0)	4 (66.7)
**Work status**				
	Not working or on maternity leave, n (%)	2 (18.2)	1 (20.0)	1 (16.7)
	Working, n (%)	9 (81.8)	4 (80.0)	5 (83.3)
	Work hours, mean (SD)	27.4 (6.7)	24.5 (5.3)	29.8 (7.2)
Language level: native speaker, n (%)		9 (81.8)	5 (100)	4 (66.7)
**Education, n (%)**				
	High school diploma	3 (27.3)	0 (0)	3 (50.0)
	College or university degree	8 (72.7)	5 (100)	3 (50.0)
Treatment for gestational diabetes mellitus: insulin, n (%)		7 (63.6)	2 (40.0)	5 (83.3)
iPhone usage: >10 times per day, n (%)		9 (81.8)	4 (80.0)	5 (83.3)
**Health app usage, n (%)**				
	Current	2 (18.2)	2 (40.0)	0 (0)
	Previous	5 (45.5)	3 (60.0)	2 (33.3)
	Never	4 (36.4)	0 (0)	4 (66.7)
**Gadget usage, n (%)**				
	Current	1 (9.1)	1 (20.0)	0 (0)
	Previous	1 (9.1)	0 (0)	1 (16.7)
	Never	9 (81.8)	4 (80.0)	5 (83.3)

#### Process Measures of the Intervention

**Figure 3:**
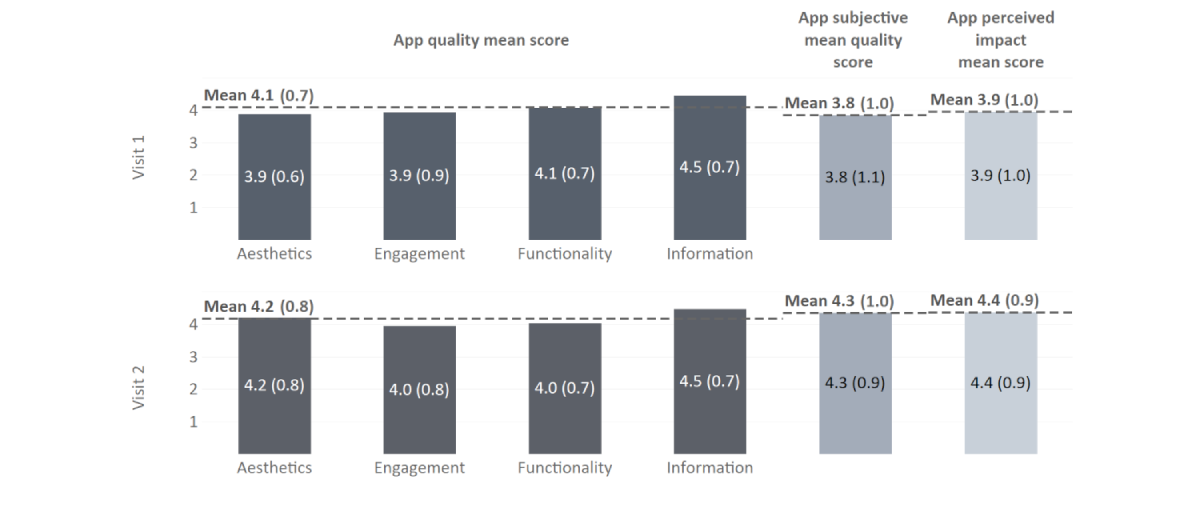
Results of the user version of the Mobile Application Rating Scale in the TRIANGLE user study stratified by visit and subscale (n=8; all values as mean [SD]).

Group 1 (n=5) tested the TRIANGLE app for approximately 1 week (9.0 days, SD 2.3 days), while group 2 (n=6) tested it for approximately 4 weeks (27.3 days, SD 3.7 days). On the SUS, participants rated the app with a mean score of 87.3 (SD 8.8) points and 87.5 (SD 10.6) out of 100 points at V1 and V2, respectively (n=11; adjective rating: “excellent”). On the uMARS, participants gave an app quality mean score of 4.1 (SD 0.7) points and 4.2 (SD 0.8) points out of a total of 5.0 points at V1 and V2, respectively (n=8; n=3 missing; Figure 3).

The highest of the 4 uMARS objective quality subscores for the TRIANGLE app was the information mean score of 4.5 (SD 0.7) points (at both V1 and V2), while the lowest subscore was the engagement mean score of 3.9 (SD 0.9) points at V1 and 4.0 (SD 0.8) points at V2 (Figure 3). The app subjective quality mean score increased from 3.8 (SD 1.1) points at V1 to 4.3 (SD 0.9) points at V2. Similarly, the perceived impact mean score increased from 3.9 (SD 1.0) points at V1 to 4.4 (SD 0.9) points at V2.

**Figure 4:**
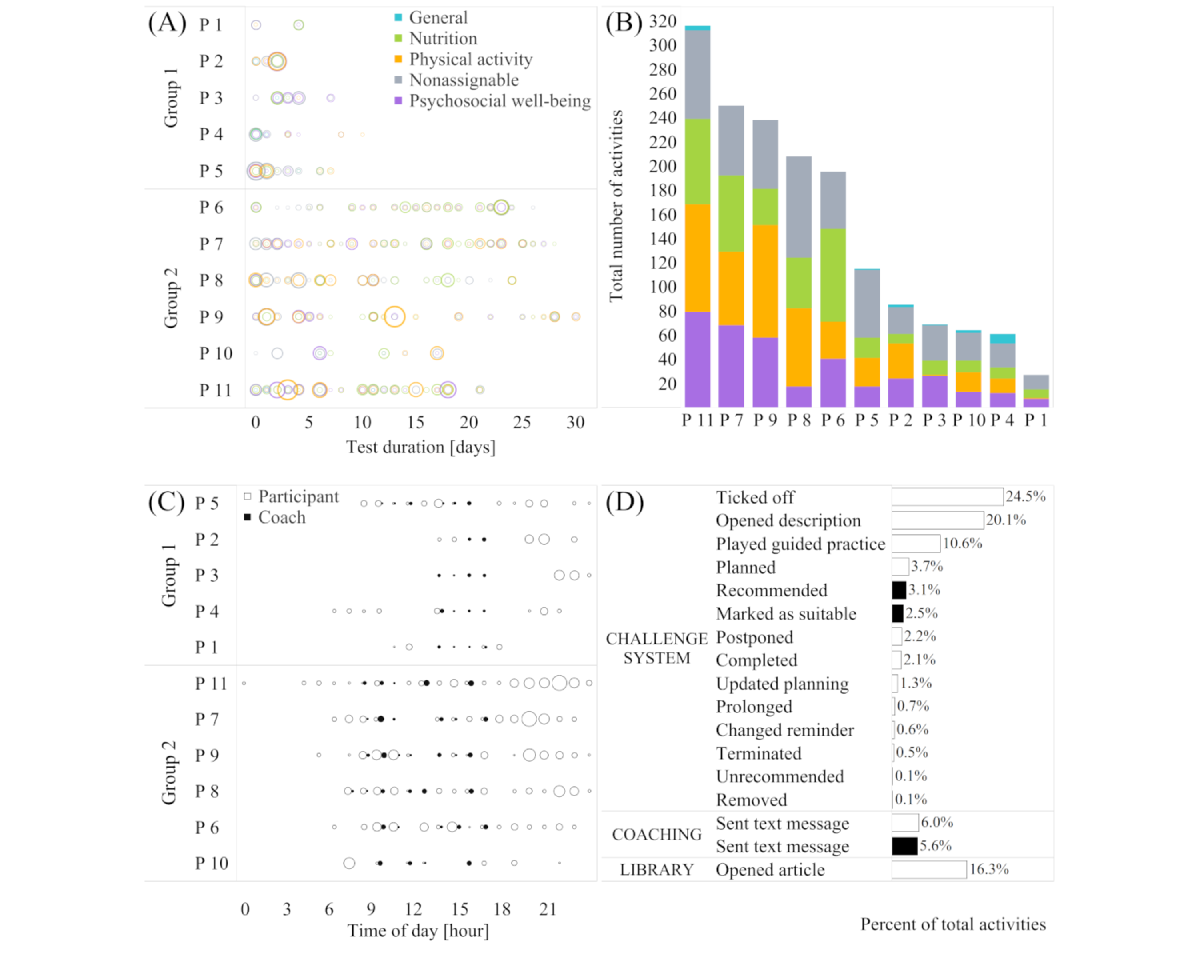
App usage in the TRIANGLE user study (n=11; group 1: n=5; group 2: n=6). (A) Number of app activities per participant over time stratified by theme; 1 circle per active day, with circle size reflecting the number of activities. (B) Total number of app activities per participant stratified by theme. (C) Number of app activities per participant and time of day; 1 circle per active hour, with circle size reflecting the number of activities. (D) Percent of app activities per subfeature. P: participant.

Most participants tested the TRIANGLE app on a regular basis, some almost daily (Figure 4A). App activity in the given test period varied by participant (Figure 4A), yet each participant tested each lifestyle area (Figure 4B). The app was used at almost any time of the day, especially in the evening, with a nocturnal pause from midnight to 4 AM (Figure 4C). Coaching times were restricted to between 7 AM and 4 PM on weekdays. Participants primarily used the challenge system by ticking off challenges and opening challenge descriptions (Figure 4D). These activities were followed by opened library articles and played guided practices. The coach showed a low activity in comparison with participants and primarily sent text messages.

### Qualitative Measures of the Intervention

Overall, participants gave positive feedback on the TRIANGLE app during the think-alouds with semistructured interviews. Participants used the app intuitively, except for 1 participant who showed initial problems with the swiping gesture. Further, participants considered the 3 core features easy to use and helpful. The combined data from the think-alouds and semistructured interviews showed 7 themes in our exploratory analysis—onboarding process (Table 4), navigation bar, challenge system, coaching, library, settings, and other feedback—with positive and negative statements for each theme. All positive statements supported the current format of the intervention.

**Table 4:** Exemplary analysis of the think-alouds with semistructured interviews for the onboarding process in the TRIANGLE user study.

Onboarding subthemes and statements from participants			Number of participants (N=11)
**Introductory video overall**			
	**Positive statements**		
		Acceptable length	9
		Good overall	1
		Answers all relevant questions	1
		Good explanation of the features	1
	**Negative statements**		
		Static image at the beginning is confusing	6
		Nonprofessional speaker	1
		Male voice for female app	1
		Marginally lengthy	1
		Too little animation	1
		Should be available at any time	1
**Introductory video content**			
	**Positive statements**		
		Contains everything important	3
		Informative value	2
		Stresses the essential	2
		Very good content	1
		Acceptable content	1
	**Negative statements**		
		Missing explanation of successful week algorithm	1
		Missing specifications of fitness tracking	1
		Marginally dense content for length	1
		Too technical	1
		Requires multiple viewings	1
		Intervention procedure not entirely clear	1
		Missing specification of coaching response time	1
**Introductory video layout**			
	**Positive statements**		
		Clear overview	1
		Nice layout	1
		Good	1
	**Negative statements**		
		Navigation not clear enough	2
		Physical fitness color not “yellow,” rather “orange”	1
		Color themes not explained in right order	1
**Chat screen as start page after the video**			
	**Positive statements**		
		Nice welcome	5
		Looks familiar	1
		Positively surprised	1
		Personal	1
		Pleasantly simple	1
	**Negative statements**		
		Not suitable	2
		Confusing	2
**First impression app**			
	**Positive statements**		
		Appears like regular app	1
		Nice overall layout	1
		Pleasantly simple	1
	**Negative statements**		
		Empty home screen before adding challenges is confusing	1

To improve the TRIANGLE app, we phrased crucial negative statements as problem descriptions and formulated the resulting changes (Table 5). We prioritized refinements and revisions of the TRIANGLE intervention based on the frequency and relevance of user feedback and the feasibility within this funding period. The main features of the TRIANGLE app remained the same before and after the pretest (Figure 2, post-testing). Additional changes to the TRIANGLE app besides those listed in Table 5 included a revised introductory video; a recipe section; more specific content for the late postpartum phase, such as pelvic floor training; an information section about the app, colored headings and subheadings per lifestyle area; and a revised paper-and-pencil questionnaire. Based on these adaptations, we revised and produced all of the TRIANGLE program materials.

**Table 5:** Exemplary problem descriptions and resulting changes to the TRIANGLE app after the TRIANGLE user study.

Problem descriptions		Resulting changes
**Navigation bar**		
	Calendar icon raised user expectations of a calendar with similar interactive subfeatures to the iPhone calendar	Progress graph icon for the progress visualization of challenges
	Closed-book icon raised user expectations of an interactive note pad	Open-book icon for the library
**Challenge system**		
	Users did not immediately notice newly available challenges	Colored number of new challenges
	Users were confused by the diverging order of challenges between the home screen and the weekly challenge overview	Synchronized challenge order of home screen and weekly challenge overview
	Users viewed the “postpone challenge” button as unnecessary given the “terminate challenge” button	Removed “postpone challenge” button
	Users considered the location of the challenge buttons at the end of a challenge description as impractical and complicating access to action	Restructured order of text and buttons in a challenge description, with buttons located at the beginning of an accepted challenge
	Users thought the challenge text with a question-answer structure was marginally lengthy	Reduced challenge text with a simplified chunking and advance organizer structure
	Users indicated an overuse of structuring elements such as capital letters, bold letters, and italics	Minimal use of structuring elements
	Challenge-specific: (1) Challenge 1.4.1: “Walk at least 3000 steps” did not apply to any user; (2) Challenge 6.1: “8 minutes of mindfulness” was considered too long by users; (3) Challenge 6.5.1: “Keep a sleep diary“ followed by challenge 6.5.2: “Analyze your sleep diary” meant too much work for users	Removed challenges 1.4.1, 6.1, 6.5.1, and 6.5.2 from the list of challenges
**Coaching**		
	Users did not immediately notice a new coaching activity	Colored number of new coaching activities in the navigation bar
	Some users were unmotivated without a sufficient number of motivational messages	Expanded database of motivational texts, some specific to each lifestyle area
	The difference between the “cancel” button in a questionnaire and the “cancel” button for exiting the canceling procedure was not clear	Renamed second “cancel” button in questionnaire tool
	Users were confused by the automatic transfer of questionnaire results to the coach upon completion without further notice	Added button for actively sending questionnaire results to the personal coach and a notification for successful transfer

## Discussion

### Principal Findings

The goal of this project was to plan and develop one of the first smartphone app–based interventions to change cardiometabolic risk behaviors in women during the first 5 years post-GDM [14,28,92,94]. The resulting TRIANGLE intervention confirmed the value of using the systematic intervention mapping framework to plan a complex health promotion program [95]. The iterative development process led to a detailed theory- and evidence-based intervention model that was translated into the TRIANGLE app and then tested by its intended users.

### Systematic Planning and Development of the TRIANGLE Intervention

We attempted to tackle some of the limitations of previous interventions during the first few years post-GDM. One of these limitations was the use of no or a single cognitive theory and few behavior change methods [96]. The more behavior change methods a health promotion program applies, the more likely it will affect behavior [97]. Hence, we used a multitheory approach at the habit-goal interface, applying 39 behavior change methods. The applied behavior change methods addressed 11 behavioral determinants and complied with the respective parameters of each method. The TRIANGLE intervention is the first intervention to prioritize habit change for women during the first 5 years post-GDM based on evidence that most health behaviors are habitual [71] and women are susceptible to unhealthy habits during the first few years post-GDM [98].

The TRIANGLE app contains a comprehensive multimedia database of newly developed lifestyle content with evidence-based information and exercises at the habit level to meet the needs of women during the first 5 years post-GDM. The content includes psychosocial well-being along with the more common DPP goals for nutrition and physical activity [7]. This addition was suggested in recent reviews [58,99] and is neededeg to prevent postnatal depression in women post-GDM [99]. Compared with previous programs during the first 5 years post-GDM, our extensive list of 81 performance objectives distinguishes between preparatory and habitual actions. Further, our performance objectives include many subbehaviors and supportive behaviors to achieve a greater behavioral outcome. In addition, we target major health decisions and communication goals as necessary actions. Few other programs during the first few years post-GDM address postpartum topics such as pelvic floor health and sleep disruptions [98], which are included in the TRIANGLE intervention.

In addition, we considered the real-life intervention context and created a practical tool for both health care practitioners and women during the first few years post-GDM [100]. This tool has the potential for widespread implementation, low-cost intervention delivery in daily life, and human coaching by multiple health care professionals. The resulting tool also shows the importance of academia-industry cooperation in order to fulfil both standards in mHealth, as recently requested by women in the first few years post-GDM [14]. Our academia-industry cooperation led to a highly usable and accepted mHealth product with unique themes, components, scope, and sequence, while most other programs during the first 5 years post-GDM were based on the DPP model. We set up strong technical assistance to support the program engagement goal of at least 5 out of 7 days per week versus the more traditional weekly or monthly interactions [101]. Further, we translated the components of skill training, behavior change support, and knowledge transfer into a unique challenge-coaching-library feature system with flexible pacing. In contrast to the flexible pacing desired by women in the first 2 years post-GDM [11], most other programs followed fixed sequences such as the DPP curriculum [89,102]. Current programs are testing different features for smartphone-based interventions in the first year post-GDM, varying degrees of automation, and cross-links to other platforms and thus will soon provide further insights [14,28,94].

Lastly, the systematic planning and development led to a transparent description of the underlying theory of the TRIANGLE intervention to inform future intervention models, related studies, or projects. To date, such a detailed description of a behavior intervention post-GDM is missing, making it difficult to compare and identify the most successful intervention components for this target group. Hence, our model may be of value for other work groups to replicate or learn from certain decisions at each intervention mapping step.

### TRIANGLE User Study

The TRIANGLE user study demonstrated that all of the main components of the TRIANGLE intervention were accepted by the intended users (women during the first 5 years post-GDM), including a first beta version of the TRIANGLE app for the iPhone. The study confirmed the value of mixed methods usability testing [103]. The SUS ratings of participants indicated “excellent” system usability [104] of the TRIANGLE app. The uMARS quality ratings of the TRIANGLE app (ie, 4.2 out of 5.0 points at V2) lay just below the top range of uMARS quality ratings for health apps (ie, 4.3 points [105])—mainly because of the lower engagement mean score compared with the other subscores. This indicated a need to enhance engagement, yet may also be related to both the limited content and the limited coach interaction in the user study. The increase in the app perceived impact mean score after several days of app usage demonstrated that users perceived a higher influence of the app on their behavior over time, as they may have started to change their health behaviors.

Overall, a smartphone app seemed to match the needs of women during the first 5 years post-GDM because participants used the TRIANGLE app regularly at almost any time of the day throughout the different lifestyle modules. The regular overall usage of the TRIANGLE app confirmed the feasibility of our usage goal of 5 active days per week. However, just like for a similar app [93], usage varied widely between participants. Each participant used each lifestyle area and thus confirmed the value of the added psychosocial module, as suggested by a recent review [58]. The high usage of the interactive challenge system stressed the importance for women to check off completed tasks for self-monitoring [93] and to view their own progress [14] in the first years post-GDM. In contrast, coaching requirements were much lower and indicated a realistic scope for implementation in routine care, especially when considering that some coaching activities may be automated in the future.

During the think-aloud sessions, participants used the app intuitively and considered the features useful. Further, the positive user statements during the semistructured interviews confirmed the chosen features and lifestyle modules. We specified and clustered emerging usability issues and negative statements as problem statements to guide smaller adaptations before the full program production, similar to related apps [14,93]. The desire of single participants for new features will have to be quantified before changing the TRIANGLE app system. Some of these desired features for apps in the first year post-GDM are currently being tested by other work groups, such as a built-in pedometer to translate other types of physical activity into step counts [28].

### Limitations

The development of the TRIANGLE intervention using the intervention mapping approach had several limitations. The comprehensive intervention mapping approach is resource consuming, and this project offered limited human and financial resources and a tight timeline due to the fast-paced technological environment. Hence, shortcuts as suggested by the authors of intervention mapping [15] had to be taken, similar to other projects applying this approach [106,107]. These shortcuts led to limited stakeholder involvement [107], especially in the planning group [15]; the focus on individual behavior change in women post-GDM without targeting the behavior of environmental agents such as their partners [108]; BMI-based tailoring only; and a respective prioritization of performance objectives, change objectives, behavior change methods, and practical applications. Lastly, we conducted intervention mapping steps 1 to 4 only and thus lack the planning and data on large-scale implementation (step 5), evaluation (step 6), and cost-effectiveness of the TRIANGLE intervention.

The user study sample was rather homogenous and may exhibit a socioeconomic bias due to its inclusion of iPhone users only and women with a high education level who were mostly native speakers. Women during the first 5 years post-GDM participated voluntarily, which may have preselected those with a higher motivation to test the TRIANGLE app. Therefore, the sample’s usage of the app, respective feedback, and suggested adaptations may not be representative of the priority population. In addition, the limited content available for the user study may have biased the distribution of activities per lifestyle area. Future studies on the TRIANGLE app need to address these factors and test its usability on the Android system.

### Options for Improvements of the TRIANGLE Intervention

In the scope of larger future projects, we suggest to additionally support behavior change by key environmental agents such as partners [108], health care professionals [94], or families [109] and to develop specific content for cultural subgroups that could not be addressed in this project [110]. Further, other cardiometabolic risk behaviors of women during the first 5 years post-GDM that we excluded need to be addressed, such as breastfeeding [111] and smoking [112]. In addition, new strategies associated with health benefits for women during the first 5 years post-GDM that contribute to the existing behavioral goals should be incorporated, such as intuitive eating [113] or postpartum emotional health [102]. Next, we identified other subgroups that need to receive tailored content in the future, such as those with low socioeconomic status [26]. Untapped behavior change methods may be added, such as motivational interviewing. Motivational interviewing was recently implemented in a digital lifestyle modification program [114] and is promising for future mHealth interventions. In general, the craving for more content needs to be addressed (eg, by weekly new content cycles [93]). Lastly, new features that further increase engagement with the app will have to be added and some of the coaching needs to be automated.

### Conclusions

The intervention mapping steps 1 to 4 were used to plan, develop, and pretest the digital TRIANGLE intervention to prevent cardiometabolic disease in women during the first 5 years post-GDM. This project description with a detailed intervention model extends previous programs post-GDM and may guide the development of similar complex smartphone solutions to support behavior change. The TRIANGLE intervention uses one of the first interactive smartphone apps to address individual habit changes in several lifestyle areas with diverse behavior change methods and flexible pacing. The TRIANGLE app meets academic and industrial standards as a result of a respective cooperation. A user sample from the target group accepted the novel TRIANGLE intervention, rated the TRIANGLE iOS app to be of high quality, and perceived it to have a large impact on behavior. The TRIANGLE app's core features of “challenge system,” “human coaching,” and “library” seem suitable for large-scale implementation and the overall feedback was positive. However, some additional user needs and subgroups will have to be addressed to further refine the TRIANGLE intervention. The Test TRIANGLE study (German clinical trials register DRKS00012996), a small multicenter randomized controlled clinical trial, explores whether the TRIANGLE intervention is followed and accepted by women during the first years post-GDM and whether it changes their cardiometabolic risk behaviors. After the programming of the app for the Android system, a larger clinical trial needs to assess the program’s efficacy and cost-effectiveness in a representative sample. Intervention mapping steps 5 and 6 may guide program implementation and evaluation in a routine care setting, with increased stakeholder involvement during the respective planning.

## Appendix

Multimedia Appendix 1 TRIANGLE app content per lifestyle area as tested in the user study.

Multimedia Appendix 2 Mixed methods design of the TRIANGLE user study.

Multimedia Appendix 3 Test setup of the think-aloud sessions in the TRIANGLE user study.

Multimedia Appendix 4 Specification of the TRIANGLE app user logs.

Multimedia Appendix 5 Logic model of the problem of type 2 diabetes and related cardiometabolic disturbances after gestational diabetes.

Multimedia Appendix 6 Performance objectives for behavioral outcomes.

Multimedia Appendix 7 Logic model of behavior change for cardiometabolic health after gestational diabetes.

Multimedia Appendix 8 TRIANGLE intervention modules and submodules.

## Abbreviations

DPP: diabetes prevention program
GDM: gestational diabetes mellitus
MAGDA-DPP: Mothers After Gestational Diabetes in Australia Diabetes Prevention Program
mHealth: mobile health
PPSDiab: Prediction, Prevention and Sub-classification of type 2 Diabetes
SUS: System Usability Scale
uMARS: user version of the Mobile Application Rating Scale
V1: visit 1
V2: visit 2
