# Supplementary material for: A Smartphone App (TRIANGLE) to Change Cardiometabolic Risk Behaviors in Women Following Gestational Diabetes Mellitus: Intervention Mapping Approach
Source: JMIR Mhealth Uhealth. 2021 May 11;9(5):e26163. doi: 10.2196/26163 (PMC8150415; doi:10.2196/26163)
Supplement: Multimedia Appendix 1 [file mhealth_v9i5e26163_app1.docx]

Multimedia Appendix 1: *TRIANGLE* app content per lifestyle area as tested in the user study. Part 1. Physical activity.

| Questionnaire | Challenge | Library articles |
| --- | --- | --- |
|  | Challenge 1.1 “Monitor daily step count”, automatically followed by challenge 1.2 “Calculate the average daily step count of this week “ |  |
| In-app question on daily average step count of one week by personal coach | *If walking less than 2,500 steps a day:* Challenge 1.4.1 “Walk at least 3,000 steps”, automatically followed by challenges 1.4.2, 1.4.3, and 1.4.4  *If walking between 2,500 and 5,000 steps a day:* Challenge 1.4.2 “Walk at least 5,500 steps”, automatically followed by challenges 1.4.3, and 1.4.4  *If walking between 5,000 and 7,500 steps a day:* Challenge 1.4.3 “Walk at least 8,000 steps”, automatically followed by challenge 1.4.4  *If walking between 7,500 and 9,500 steps a day:* Challenge 1.4.4 “Walk at least 10,000 steps” | Daily physical activity basics  Daily biking basics (video tutorial)  Tips on posture in daily life and sports (4 video tutorials)  Walking basics (video tutorial)  Jogging basics (video tutorial) |
|  | Challenge 1.7 “Conduct the fitness self-test” (5 guided practice videos) | Physical fitness basics  Cardio training basics |
|  | Challenge 1.10 “Daily 10-minute training” (12 guided practice videos, including warmup and cooldown) | Resistance training basics  Ideal training intensity  Individual adaptation of training intensity (4 video tutorials)  Muscle ache (2 video tutorials)  The need for active regeneration  (2 video tutorials)  Warmup and cooldown basics (3 video tutorials)  Breathing support during resistance training (video tutorial)  The right technique for basic resistance exercises (7 video tutorials) |

Multimedia Appendix 1 (continued): Part 2. Nutrition

| Questionnaire | Challenge | Library articles |
| --- | --- | --- |
| *If habitual breakfast cereal eater (as assessed in initial paper and pencil lifestyle questionnaire):* In-app questionnaire about habitual muesli composition | *If less than one quarter of whole grain:*  Challenge 2.11.1 “Have your muesli with one quarter of whole grain”  *If less than one quarter of lean protein:*  Challenge 2.11.2 “Have your muesli with one quarter of lean protein”  *If less than one half of fresh fruit:*  Challenge 2.11.3 “Have your muesli with one half of fresh fruit”  *If one or two components are not met:*  Challenge 2.11.4 “Have your ideal muesli”  *If none of the components are met:*  Challenge 2.11.1, automatically followed by 2.11.2, 2.11.3, and 2.11.4 | Nutrition basics  Eating basics  Basic grocery shopping list |
|  | Challenge 2.11.5 “Have your bread meal with one quarter of whole grain”  Challenge 2.11.6 “Have your bread meal with one quarter of lean protein”  Challenge 2.11.7 “Have your bread meal with one half of fresh fruit or vegetable”  Challenge 2.11.8 “Have your ideal bread meal”  Challenge 2.11.5, automatically followed by 2.11.6, 2.11.7, and 2.11.8 | Tips for an optimal meal preparation  Cooking basics  Healthy snacking |
| Initial paper and pencil lifestyle questions include drinking behavior | *If less than 1.5 l of pure water OR predominantly drinks other than water, black coffee or plain tea:*  Challenge 5.12.1 “Find your perfect thirst quencher”  Challenge 5.12.2 “Drink at least 1.5 l of pure water a day”  Challenge 5.12.3 “Drink at least 1.5 l of pure water or plain herbal tea a day and limit other drinks” | Beverage basics  Optimal drinking  Beverages – the exceptions |

Multimedia Appendix 1 (continued): Part 3. Psychosocial wellbeing

| Questionnaire | Challenge | Library articles |
| --- | --- | --- |
| In-app sleep questionnaire | *If current sleep problems are caused by infant’s sleep problems:*  Challenge 6.4 “Implement sleep enhancing recommendations for infants.”  *If current sleep problems independent from infant:*  Challenge 6.5.1 “Keep a sleep diary”, automatically followed by challenge 6.5.2 “Analyze your sleep diary”, and by challenge 6.5.3 “Implement sleep enhancing recommendations for own sleep.” | Basics for inner balance  Sleep tips for me  Sleep tips for my child  Relaxation basics  Four types of relaxation and suitable experiences of relaxation |
| Initial paper and pencil lifestyle questions include mindfulness | Challenge 6.1 “Eight minutes of mindfulness”, automatically followed by challenge 6.2 “Five minutes of mindfulness“, challenge 6.3 “Three minutes of mindfulness“, and challenge 6.4 “Three mindful moments per day” | Mindfulness basics  Tips for mindfulness practice  Mindfulness exercises (6 guided practice audios)  Optimism basics  Five pessimistic and optimistic thinking patterns  Self-efficacy basics |
