# Supplementary material for: A Smartphone App (TRIANGLE) to Change Cardiometabolic Risk Behaviors in Women Following Gestational Diabetes Mellitus: Intervention Mapping Approach
Source: JMIR Mhealth Uhealth. 2021 May 11;9(5):e26163. doi: 10.2196/26163 (PMC8150415; doi:10.2196/26163)
Supplement: Multimedia Appendix 2 [file mhealth_v9i5e26163_app2.docx]

Multimedia Appendix 2: Mixed methods design of the *TRIANGLE* user study

|  | Visit 1 | App testing | Visit 2 |
| --- | --- | --- | --- |
|  | 3-60 months post-GDM | Group 1: for 1 week  Group 2: for 4 weeks | Group 1: after 1 week  Group 2: after 4 weeks |
| Verification of in- and exclusion criteria | X (prior to visit 1) |  |  |
| Informed consent | X |  |  |
| Anamnesis | X |  |  |
| System Usability Scale (SUS) and user Mobile Application Rating Scale (uMARS) | X |  | X |
| User registration app | X |  |  |
| In-app questionnaires |  | X (optional) |  |
| User logs | X | X | X |
| One-on-one “think aloud” with subsequent semi-structured interview | X |  | X |

GDM = gestational diabetes mellitus
