# Supplementary figures and images for: A Smartphone App (TRIANGLE) to Change Cardiometabolic Risk Behaviors in Women Following Gestational Diabetes Mellitus: Intervention Mapping Approach
Source: JMIR Mhealth Uhealth. 2021 May 11;9(5):e26163. doi: 10.2196/26163 (PMC8150415; doi:10.2196/26163)

Multimedia Appendix 3: Test set-up of the think aloud sessions in the *TRIANGLE* user study

| 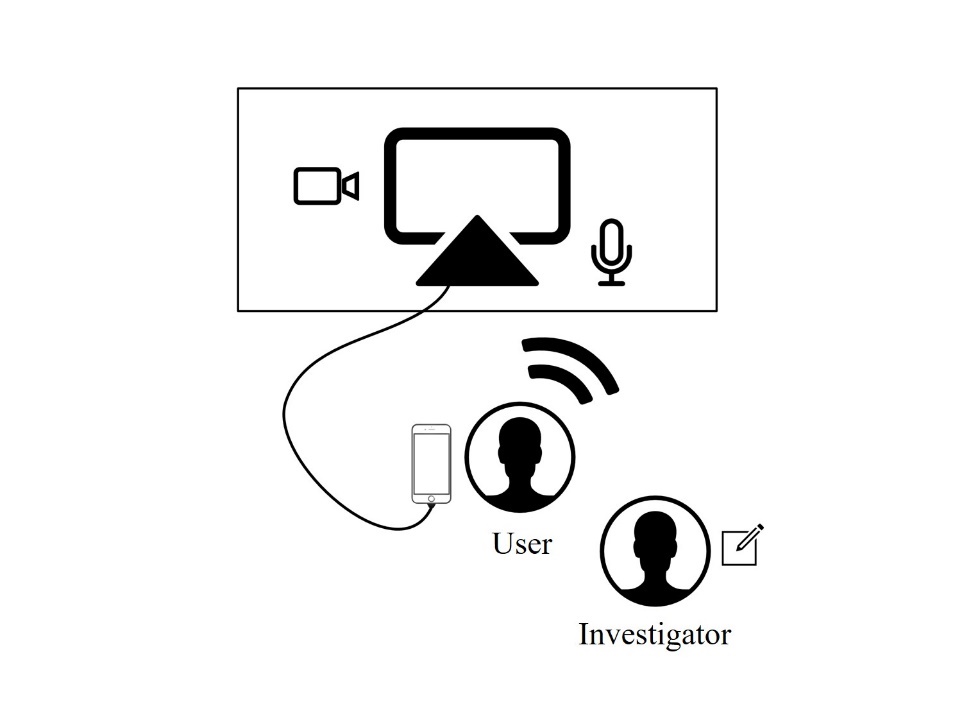 |
| --- |

Supplement: Multimedia Appendix 3 [file mhealth_v9i5e26163_app3.docx]
