# Supplementary material for: A Smartphone App (TRIANGLE) to Change Cardiometabolic Risk Behaviors in Women Following Gestational Diabetes Mellitus: Intervention Mapping Approach
Source: JMIR Mhealth Uhealth. 2021 May 11;9(5):e26163. doi: 10.2196/26163 (PMC8150415; doi:10.2196/26163)
Supplement: Multimedia Appendix 4 [file mhealth_v9i5e26163_app4.docx]

Multimedia Appendix 4: Specification of the *TRIANGLE* app user logs

| Technical core feature | Actor | Motion data of technical sub-feature with time stamp |
| --- | --- | --- |
| Challenge system | Coach | - (Un-) marked challenge as suitable for user - (Un-) recommended challenge for user |
|  | User | - Opened challenge information - Planned challenge - Updated challenge planning - Played video or audio file in challenge - Ticked off challenge (retrospectively) - Undo ticked off challenge (retrospectively) - Terminated challenge - Prolonged challenge - Postponed challenge - Completed challenge - Changed reminders |
| Coaching | Coach | - Sent text message to user - Sent questionnaire to user |
|  | User | - Sent text message to coach - Completed questionnaire |
| Library | User | - Opened library article - Played video or audio file in library article |
| Other | User | - Registered with individual code |
