# Supplementary material for: A Smartphone App (TRIANGLE) to Change Cardiometabolic Risk Behaviors in Women Following Gestational Diabetes Mellitus: Intervention Mapping Approach
Source: JMIR Mhealth Uhealth. 2021 May 11;9(5):e26163. doi: 10.2196/26163 (PMC8150415; doi:10.2196/26163)
Supplement: Multimedia Appendix 5 [file mhealth_v9i5e26163_app5.docx]

Multimedia Appendix 5: Logic model of the problem for type 2 diabetes and related cardiometabolic disturbances post gestational diabetes

| 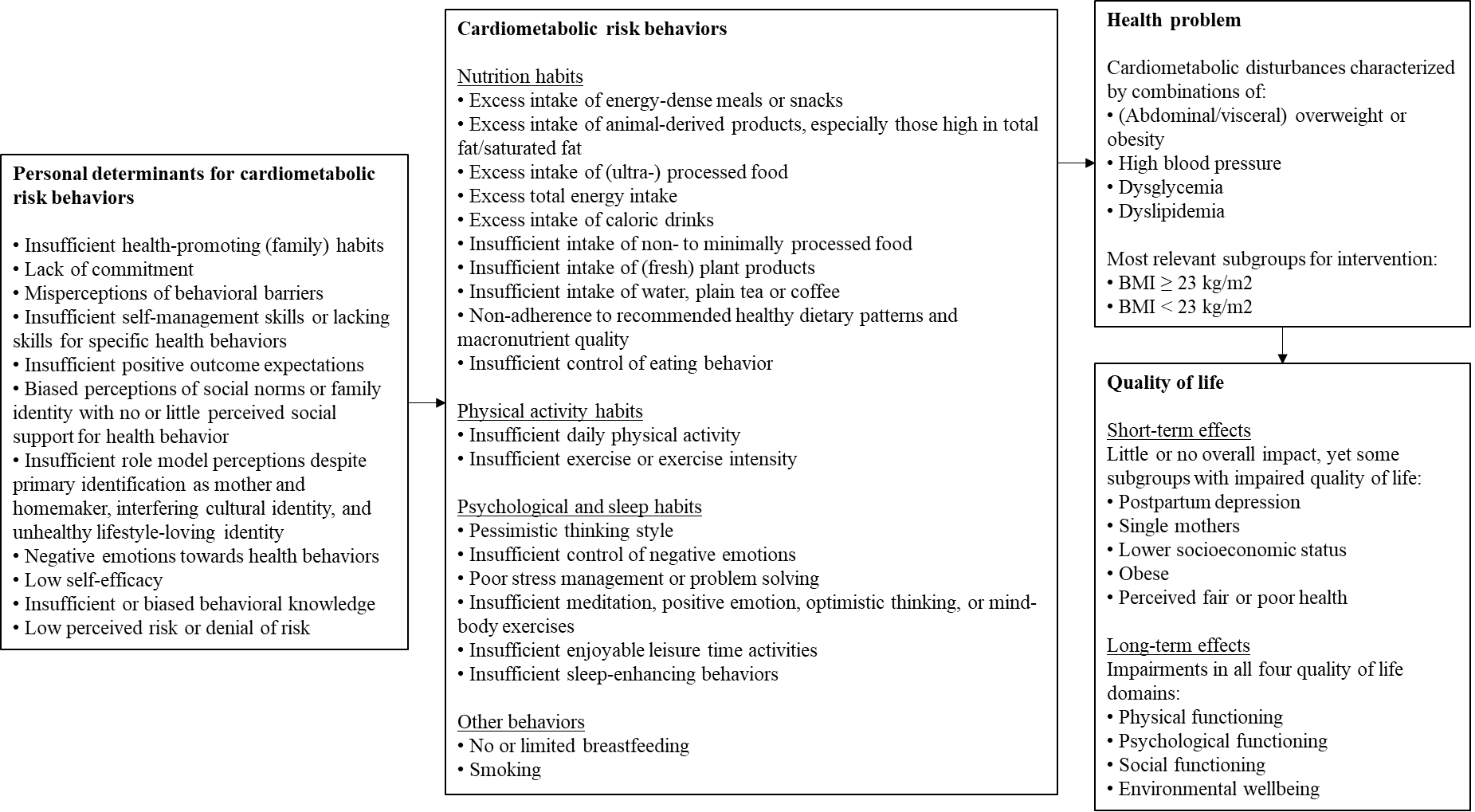 |
| --- |
