# Supplementary material for: A Smartphone App (TRIANGLE) to Change Cardiometabolic Risk Behaviors in Women Following Gestational Diabetes Mellitus: Intervention Mapping Approach
Source: JMIR Mhealth Uhealth. 2021 May 11;9(5):e26163. doi: 10.2196/26163 (PMC8150415; doi:10.2196/26163)
Supplement: Multimedia Appendix 6 [file mhealth_v9i5e26163_app6.docx]

Multimedia Appendix 6: Performance objectives for behavioral outcomes

| Behavioral outcomes (BO) | Associated performance objectives (PO)  *Woman post-GDM will:* |
| --- | --- |
| BO.2. Consume at least 15 g of dietary fiber per 1,000 kcal energy intake. | PO.2.1. Decide to gradually eat more plant-based non- to minimally processed food with sufficient amounts of whole grain products, legumes, nuts, seeds, fruits, and vegetables to enhance dietary fiber intake to at least 15 g per 1,000 kcal per day.  PO.2.2. Communicate health goal to the family to gradually increase dietary fiber intake by eating more whole grain products, legumes, nuts, seeds, fresh fruits, and fresh vegetables.  PO.2.3. Establish a healthy family recipe book with at least 30 favorite recipes according to the healthy meal model with a quarter whole grain, a quarter preferably plant-based lean protein, and half fresh fruit or vegetable.  PO.2.4. Carefully read food labels for buying or consumption decisions.  PO.2.5. Remove unhealthy and ultra-processed food products from home food supplies.  PO.2.6. Keep predominantly plant-based non-/minimally processed home food supplies (whole grain products, legumes, nuts, and seeds).  PO.2.7. Establish a weekly family meal planning based on the healthy family recipe book.  PO.2.8. Write a grocery list for the weekly meal plan, add healthy snacks, and separate a smaller mid-weekly list for fresh products.  PO.2.9. Establish healthy grocery shopping strictly according to the grocery list.  PO.2.10. Maintain a healthy cooking routine according to the weekly meal plan.  PO.2.11. Depending on the starting point, gradually increase eating main meals by the healthy meal model, with a quarter whole grain, a quarter preferably plant-based lean protein, and half fresh fruit or vegetable.  PO.2.12. Eat at least two fist size portions of whole grain products a day.  PO.2.13. Eat at least one fist size portion of legumes a day.  PO.2.14. Eat two hand size portions of fresh fruit a day.  PO.2.15. Eat at least three hand size portions of fresh vegetable a day. |
| BO.3. Consume less than 30% of total energy intake from fat. | PO.3.1. Communicate health goal to the family to gradually limit or replace high-fat food products for a total fat intake of less than 30% of total energy intake.  PO.3.2. Get familiar with naturally low-fat food products and meals.  PO.3.3. Gradually limit or replace high-fat food products to reduce daily fat intake to less than 30% of total energy intake – especially those derived from animal sources and ultra-processed food.  PO.3.4. Establish a low-fat nutrient-protective meal preparation.  PO.3.5. Generously use fresh herbs and spices. |

BO = behavioral outcome, GDM = gestational diabetes mellitus, PO = performance objective

Multimedia Appendix 6 (continued)

| Behavioral outcomes (BO) | Associated performance objectives (PO)  *Woman post-GDM will:* |
| --- | --- |
| BO.4. Consume less than 10% of total energy intake from saturated fat. | PO.4.1. Communicate health goal to the family to gradually limit or replace food products high in saturated fat.  PO.4.2. Get familiar with food products and meals naturally low in saturated fat and trans-fat.  PO.4.3. Gradually limit or replace food products high in saturated fat to reduce daily saturated fat intake to less than 10% of total energy intake – especially those derived from animal sources and ultra-processed food products. |
| BO.5. Reduce body weight by at least 5% if BMI is 23 kg/m^2^ or higher, maintain body weight if BMI is less than 23 kg/m^2^. | PO.5.1. Keep a food journal to explore own nutrition patterns (personal cues for food intake, eating times, eating environment, etc.).  PO.5.2. Investigate what to change in own eating situations to select healthier food, eat more mindfully, and to fully enjoy the food.  PO.5.3. Create pleasant eating situations in daily life.  PO.5.4. Write a personal list with distractions for food cravings with detailed descriptions how to react in particular circumstances.  PO.5.5. Implement distractions when a specific food craving emerges.  PO.5.6. Eat when hungry, not when thirsty, having an appetite or other needs, and learn to distinguish these sensations.  PO.5.7. If BMI is 23 kg/m2 or higher: Avoid added sugar and sweeteners for at least one week.  PO.5.8. If BMI is less than 23 kg/m2: Limit added sugar intake to less than 25 g per day.  PO.5.9. If habitual unhealthy snacking: Get familiar with healthy snacks based on fresh fruit and vegetable.  PO.5.10. If habitual unhealthy snacking: Snack fresh fruit or vegetable on the go or at home.  PO.5.11. If water intake below 1.5 l: Taste naturally flavored drinking water.  PO.5.12. Drink at least 1.5 - 2 l of water or pure herbal tea a day, and limit other drinks to maximum four cups of plain black/green tea or black coffee and one glass of a 3:1 mixed water with juice.  PO.5.13. If eating irregularly every day: Keep a meal rhythm.  PO.5.14. If frequent snacking: Limit snacking to two healthy snacks between meals per day.  PO.5.15. If BMI is 23 kg/m2 or higher: Eat one regular sized portion per meal.  PO.5.16. If BMI is less than 23 kg/m2: Replace energy-dense and ultra-processed food products with healthier alternatives.  PO.5.17. If overnight fasting habit of less than 12 hours: Gradually extend the overnight fast to at least twelve to sixteen hours between the last and the first food or drink intake other than water. Find out which time slot best aligns with own schedule and family life.  PO.5.18. If BMI is 23 kg/m2 or higher: Lose one to two kilos of body weight per month until own weight goal is reached.  PO.5.19. Remain flexible in controlling eating behavior. Commit to a healthy nutrition on a habitual basis, yet stay flexible for special occasions and do not ban entire food categories. Balance exceptions in the next meal or snack, the latest on the next day. |

BMI = body mass index, BO = behavioral outcome, GDM = gestational diabetes mellitus, PO = performance objective

Multimedia Appendix 6 (continued)

| Behavioral outcomes (BO) | Associated performance objectives (PO)  *Woman post-GDM will:* |
| --- | --- |
| BO.6. Increase psychosocial wellbeing and sleep, decrease stress perception. | PO.6.1. Practice mindfulness to be fully present during daily experiences. Start with scheduled daily five-minute mindfulness exercises and gradually lower the exercise time to three minutes a day and then to at least three moments a day.  PO.6.2. Get familiar with different types of recreational activities and select favorite three.  PO.6.3. Maintain a weekly recreational activity routine. Start with one scheduled recreational activity per week and gradually increase to three scheduled recreational activities per week.  PO.6.4. Enhance infant’s sleep with list of recommended actions.  PO.6.5. Enhance own sleep with list of recommended actions.  PO.6.6. Maintain a daily progressive muscle relaxation routine. Start with a scheduled daily 18-minute progressive muscle relaxation exercise and gradually lower the exercise time to seven minutes and then to several short full muscle relaxation moments during the day.  PO.6.7. Practice gratitude by reviewing the positive things happening on a regular day. Start with two items per day and gradually increase to four items.  PO.6.8. Identify the most predominant one or two automatic negative thoughts in own thinking style and select corresponding positive thoughts to replace them with.  PO.6.9. Notice automatic negative thoughts, acknowledge them and replace them with the according positive thoughts.  PO.6.10. Get familiar with emotional positivity and learn how to strengthen positive emotions in daily life. Start by focusing on one positive emotion. Think about three situations in the past when the chosen emotion was present. Think about three concrete situations in the upcoming week to feel that chosen emotion, imagine them in detail and write them down.  PO.6.11. Establish a weekly routine to strengthen positive emotions.  PO.6.12. Get familiar with seven steps for problem solving and learn to apply them.  PO.6.13. Establish a problem-solving routine using seven recommended steps. Start with a particular problem. Describe the problem, then the target state in as much detail as possible. Brainstorm at least three different ways to reach the target state, then choose the most achievable path for the upcoming four weeks. Break the selected path down into milestones and schedule them. Work towards each milestone. Once the steps are completed, evaluate each step.  PO.6.14. Establish a prioritization routine of current and upcoming activities with a decision matrix.  PO.6.15. Get familiar with character strengths and identify the top three personal strengths.  PO.6.16. Focus on the top three personal strengths in daily life. |

BO = behavioral outcome, GDM = gestational diabetes mellitus, PO = performance objective

Multimedia Appendix 6 (continued)

| Behavioral outcomes (BO) | Associated performance objectives (PO)  *Woman post-GDM will:* |
| --- | --- |
| BO.7. Adhere to the mHealth program and enhance self-management. | PO.7.1. Participate in mHealth program on at least five out of seven days per week.  PO.7.2. Communicate with healthcare practitioners via mHealth app.  PO.7.3. Take recommended questionnaires in mHealth app to become aware of current behaviors and to assess discrepancies between current and recommended behavior.  PO.7.4. Select and commit to own long-term health goals in mHealth app.  PO.7.5. Select and commit to health actions in mHealth app.  PO.7.6. Set reminders, schedule, or link chosen health actions to repeated contexts.  PO.7.7. Monitor own progress of health actions and health goals in mHealth app on a weekly basis.  PO.7.8. Reschedule missed or skipped health actions.  PO.7.9. Evaluate own performance in chosen health actions and health goals in mHealth app when recommended.  PO.7.10. Adapt chosen health goals and health actions when necessary. |

BO = behavioral outcome, GDM = gestational diabetes mellitus, mHealth = mobile health, PO = performance objective
