# Supplementary material for: A Smartphone App (TRIANGLE) to Change Cardiometabolic Risk Behaviors in Women Following Gestational Diabetes Mellitus: Intervention Mapping Approach
Source: JMIR Mhealth Uhealth. 2021 May 11;9(5):e26163. doi: 10.2196/26163 (PMC8150415; doi:10.2196/26163)
Supplement: Multimedia Appendix 8 [file mhealth_v9i5e26163_app8.docx]

Multimedia Appendix 8: *TRIANGLE* intervention modules and submodules

| Physical activity | Nutrition | Psychosocial wellbeing | Intervention adherence |
| --- | --- | --- | --- |
| Basics for physical activity | Basics for a healthy nutrition | Basics for psychosocial wellbeing | Basics for behavior change |
| Daily steps and activity | Drinks | Mindfulness practice | Participatory decision making |
| Disrupting long sedentary periods | Home food supplies | Recreational activities | Communication with healthcare practitioner |
| Endurance training | Grocery shopping | Progressive muscle relaxation routine | Questionnaires and self-tests |
| Resistance training | Meal planning | Gratitude practice | Notifications |
| High intensity interval training | Healthy meal composition | Optimistic thinking | Feedback |
| Flexibility training | Healthy snacks | Emotional positivity | Scheduling and reminders |
| Barriers to physical activity and how to overcome them | Meal rhythm | Problem solving | Self-monitoring |
|  | Mindful eating | Prioritization | Frequently asked questions |
|  | Flexible control | Character strengths |  |
|  | Eating cues | Sleep |  |
|  | Added sugar limit |  |  |
|  | Non to minimally processed food |  |  |
|  | Sufficient dietary fiber |  |  |
|  | Extended overnight fast |  |  |
|  | Caloric restriction |  |  |
|  | Healthy alternatives |  |  |
|  | Portion size |  |  |
|  | Barriers to a healthy nutrition and how to overcome them |  |  |
